# Supplementary material for: Dynamic transcriptomic profiles of zebrafish gills in response to zinc supplementation
Source: BMC Genomics. 2010 Oct 11;11:553. doi: 10.1186/1471-2164-11-553 (PMC3091702; doi:10.1186/1471-2164-11-553)
Supplement: Additional file 2 — Interactive Direct Interaction Network representing the molecular interactions between zinc, copper, iron, calcium and proteins encoded by transcripts changed by zinc supplementation. Mini web-site containing index.html and hyperlinked pages in subdirectory describing a Direct Interaction Network automatically generated based on curated interactions contained within the proprietary PathwayArchitect database. Ovals represent proteins and the circles symbolize metal ions. Objects are coloured by their abundance in zebrafish at the time-point they were significantly different from the control is a scale from -4 fold (dark green) to +4 fold (dark red). Where significant differences were found at more than one time-point, the colour overlay shows expression at the first instance. Dark blue squares denote 'binding', and light blue squares 'expression'; green squares stand for 'regulation', green diamonds for 'metabolism', and green circles for 'promoter binding'. Arrow heads indicate directionality of the interaction where annotated. All nodes and edges can be further interrogated by selecting the relative area of the image. [file 1471-2164-11-553-S2.zip › PathwayArchitect Zn xs DIN/688647.html]

# REGULATION:

|  |  |
| --- | --- |
| Type | REGULATION |
| Effect | Negative |


---

|  |  |
| --- | --- |
| Score | 0 |


---

|  |  |
| --- | --- |
| Reference Count | 10 |


---

|  |  |
| --- | --- |
| Mechanism | Unknown |


---

|  |  |
| --- | --- |
| Reference:0 || Sentence | "Incubating with both zinc and TEPA reduced copper levels relative to the control, but did not reduce Cu/Zn-SOD activity to the same extent as TEPA alone." |
| PMID | 1333521 |
| Year | 1992 |
| Species | Human |
| Journal | J Nutr |
| RefScore | 0 |
| Source | PArchNLP |
  |
|  |  |


---

|  |  |
| --- | --- |
 Reference:1 || Sentence | "After zinc supplementation, serum copper concentration decreased but there was no change in the ratio of low-density-lipoprotein cholesterol to high-density-lipoprotein cholesterol." |
| PMID | 8460613 |
| Year | 1993 |
| Species | Human |
| Journal | Am J Clin Nutr |
| RefScore | 0 |
| Source | PArchNLP |
  ||  |  |


---

|  |  |
| --- | --- |
 Reference:2 || Sentence | "The role of the NF-kappaB-mediated mechanism in induction or prevention of chronic intlammatory, allergic, degenerative and tumor diseases by zinc, vanadium, manganese, copper, silica, iodine and other trace elements is discussed." |
| PMID | 11130849 |
| Year | 2000 |
| Species | Human |
| Journal | J Trace Elem Med Biol |
| RefScore | 1 |
| Source | PArchNLP |
  ||  |  |


---

|  |  |
| --- | --- |
 Reference:3 || Sentence | "Zinc's mechanism of action involves induction of intestinal cell metallothionein (Mt), which blocks copper absorption from the intestinal track." |
| PMID | 11585025 |
| Year | 2001 |
| Species | Human |
| Journal | Expert Opin Pharmacother |
| RefScore | 0 |
| Source | PArchNLP |
  ||  |  |


---

|  |  |
| --- | --- |
 Reference:4 || Sentence | "We observed striking elevation of manganese and, to a lesser extent, of zinc accompanied by significant reduction of copper bound to purified PrP in all sCJD variants, determined by the PrP genotype and PrP(Sc) type, combined." |
| PMID | 11579148 |
| Year | 2001 |
| Species | Human |
| Journal | J Neurochem |
| RefScore | 2 |
| Source | PArchNLP |
  ||  |  |


---

|  |  |
| --- | --- |
 Reference:5 || Sentence | "The reduction in Cu,Zn-SOD protein is likely due to a post-transcriptional mechanism as steady-state Cu,Zn-SOD mRNA levels measured by Northern hybridization were not altered by copper deficiency in any organ studied (liver, heart, and brain)." |
| PMID | 11516174 |
| Year | 2001 |
| Species | Rat |
| Journal | Arch Biochem Biophys |
| RefScore | 2 |
| Source | PArchNLP |
  ||  |  |


---

|  |  |
| --- | --- |
 Reference:6 || Sentence | "Plasma and tissue copper, the activities of plasma ceruloplasmin ferroxidase and erythrocyte Cu,Zn-superoxide dismutase, hematocrits, and serum cholesterol and triglyceride were all decreased (p less than 0.05) and relative cardiac mass was increased (p less than 0.05) by severe dietary copper deficiency." |
| PMID | 2360543 |
| Year | 1990 |
| Species | Rat |
| Journal | Am J Clin Nutr |
| RefScore | 1 |
| Source | PArchNLP |
  ||  |  |


---

|  |  |
| --- | --- |
 Reference:7 || Sentence | We present 3 cases of zinc-induced copper deficiency in which the diagnosis first was suggested on the basis of bone marrow examination. |
| PMID | 15762288 |
| Year | 2005 |
| Species | Human |
| Journal | Am J Clin Pathol |
| RefScore | 0 |
| Source | PArchNLP |
  ||  |  |


---

|  |  |
| --- | --- |
 Reference:8 || Sentence | However, large amounts of zinc competitively inhibit copper absorption, which inhibits the copper-dependent ceruloplasmin, and can cause a lethal anemia. |
| PMID | 15814196 |
| Year | 2005 |
| Species | Mouse |
| Journal | Neurosci Lett |
| RefScore | 1 |
| Source | PArchNLP |
  ||  |  |


---

|  |  |
| --- | --- |
 Reference:9 || Sentence | Studies have shown that (i) the olfactory lobe is normally enriched in carnosine and zinc, (ii) carnosine can suppress copper and zinc toxicity in olfactory neurones, (iii) olfactory dysfunction is often associated with neurodegenerative conditions and (iv) elevated levels of zinc are found in brains of Alzheimer's patients. |
| PMID | 16034682 |
| Year | 2005 |
| Species | Human |
| Journal | Biogerontology |
| RefScore | 2 |
| Source | PArchNLP |
  |


---

|  |  |
| --- | --- |
